# A Phospholipid Profile at 4 Months Predicts the Onset of Celiac Disease in at-Risk Infants

**DOI:** 10.1038/s41598-019-50735-7

**Published:** 2019-10-04

**Authors:** R. Auricchio, M. Galatola, D. Cielo, A. Amoresano, M. Caterino, E. De Vita, A. Illiano, R. Troncone, L. Greco, M. Ruoppolo

**Affiliations:** 1 Department of Translational Medical Sciences, University of Naples “Federico II”, Napoli, Italy; 2 European Laboratory for the Investigation of Food Induced Diseases (ELFID), University of Naples “Federico II”, Napoli, Italy; 3 Department of Chemical Sciences, University of Naples “Federico II”, Napoli, Italy; 4 Department of Molecular Medicine and Medical Biotechnology, University of Naples “Federico II”, Napoli, Italy; 5 CEINGE, Biotecnonologie Avanzate s.c.ar.l., Napoli, Italy

**Keywords:** Metabolomics, Predictive markers

## Abstract

Celiac disease (CeD) is a multifactorial disease influenced by both genetic and environmental risk factors. CeD genetic components are mainly due to HLA class II genes, which account for approximately 40% of the disease heritability. The environmental factor is linked to gliadin ingestion. Despite genetic and epigenetic studies, the pathological molecular mechanism remains unclarified. The strong genetic component does not explain more than half of the hereditability; we identified several epigenetic features that contribute to the understanding of the missing hereditability. The lipid profile of infants has been proposed as a potential biomarker of CeD metabolism that can be measured before they exhibit developmental disorders and clinical symptoms. We suggest that the state of the host is a main factor for the abnormal immune response to gluten. Long before any exposure to the offending agent or any production of specific antibodies, several molecular mechanisms are differentially expressed in infants who will develop CeD compared to their peers matched for the same genetic profile. The present study explored the serum phospholipid profile of a group of infants at risk for celiac disease, followed up to 8 years to monitor the onset of CeD. We compared 30 patients who developed the disease with 20 age- and sex-matched peers with similar genetic profiles who did not develop the disease within 8 years. Serum phospholipids were analysed at 4 months, before exposure to gluten, and at 12 months of age, when none showed any marker of disease. In the 30 CeD patients, we also analysed the serum at the time of diagnosis (>24 months). The serum phospholipid profile was fairly constant across 4 and 12 months of age and, in CeD, up to 24–36 months. The phospholipid signature was dramatically different in infants who developed CeD when compared to that of control NY-CeD (Not Yet developing Celiac Disease) peers. We identified a specific serum phospholipid signature that predicts the onset of celiac disease in HLA at-risk infants years before the appearance of antibodies specific for CeD in the serum and before any clinical symptoms, even before gluten introduction into the diet at 4 months. Specifically, lysophosphatidylcholine, phosphatidylcholine, alkylacyl-phosphatidylcholine, phosphoethanolamines, phosphatidylserines, phosphatidylglycerol and phosphatidylinositol were found to be differentially represented in CeD *versus* NY-CeD. A set constituted by a limited number of alkylacyl-phosphatidylcholine and lyso-phosphatidylcholine, together with the duration of breast-feeding, allows the discrimination of infants who develop celiac disease before 8 years of age from those at a similar genetic risk who do not develop the disease. In addition to recent discovery, our paper unveiled a specifc phopholipid profile, able to discriminate infants who eventually develop celiac disease years before antibodies or clinical symptoms ensue.

## Introduction

The lipid profile of small infants has been proposed as nutritional biomarkers of the infant metabolism^1,2^. Lipidomics is now considered an appropriate tool, not only to monitor the ongoing nutritional processes but also the future development of diseases^3^. Celiac disease (CeD) is a multifactorial disease influenced by both genetic and environmental risk factors. The CeD genetic component is mainly due to HLA class II genes and accounts for approximately 40% of the disease heritability. Despite genetic and epigenetic studies, the pathological molecular mechanism remains unclarified^4,5^; in this context, metabolomic investigations could provide new ways to address the unsolved questions about CeD.

In the Cambridge Baby Growth Study^6^, a detailed lipidomic profile from dried blood spot samples of infants allowed the differentiation of infants on breast milk from those that were bottle fed. The study showed that an early growth trajectory in preterm infants is associated with the breast milk lipidome^7^.

Recently, the serum lipidome has been proposed as an accurate predictor of islet autoimmunity and type I diabetes (T1D)^8^: the cord-blood phospholipid level seems to be a potential predictor of disease. Phospholipids were found to be significantly decreased at birth in children later diagnosed with type I diabetes by 4 years of age^9^. Subsequently, in 2018, Oresic and his group^10^ compared 40 children who progressed to T1D, 40 children who developed a single islet autoantibody but did not progress to T1D during follow up, and 40 matched controls. At 3 months of age, children who developed diabetes already showed a marked and constant downregulation of triacylglycerol and phosphatidilcholines. In addition, the same research group identified a specific signature of triacylglycerol and phosphatidilcholines, which predicted the onset of type 2 diabetes in a Finnish adult population^11^.

Oresic again recently explored the plasma lipidome in twenty-three children who progressed to CeD at a mean age of 4.8 years. They showed increased amounts of triacylglycerols (TGs) of low carbon number and double bonds count and a decreased level of phosphatidylcholines by age 3 months as compared to controls. These differences were exacerbated with age but were not observed at birth (cord blood)^12^. In 2016 the exploration of a preliminary sample of infants who developed CeD within the PREVENT-CD study was unable to identify a metabolic signature associated to the development of the disease^13^.

There is more than one reason to explore the phospholipid signature of infants who have a higher, more than ten times, risk to develop celiac disease due to the presence of at-risk HLA DQ2/DQ8 haplotype within a family with a celiac proband. A cohort of 256 new-borns from families with one celiac proband was enrolled and followed up over 8 years by a scheduled surveillance system, monitoring the health of the child and the production of anti-transglutaminase antibodies (anti-TG2). We hypothesized that the development of celiac disease in a child from a family with a proband case might be predicted long before any production of anti-TG2 antibodies or any clinical symptoms. We were able to identify a specific gene expression signature that could predict the outcome in the majority of infants in the first year of life, long before any production of antibodies^14^. This result reinforced the need to look for early predictors of outcomes in this cohort. Aim of this paper is to investigate lipidomics in order to elucidate the metabolic signature, that, associated to genetics and epigenetics features helps to identify the CeD infant phenotype in very early age in at risk children. The most predominant mammalian cellular lipidomic components as lyso-phosphatidylcholine (LPC), phosphatidylcholine (PC), alkylacyl-phosphatidylcholine (PC-O), phosphatidyletanolamine (PE), phosphatidylglycerol (PG), phosphatidylinositol (PI), phosphatidylserine (PS) groups were monitored together along with breast-feeding duration. A set constituted by alkylacyl-phosphatidylcholine along with duration of breast-feeding allowed us to discriminate infants who developed celiac disease before 6 years of age from those, at a similar genetic risk, who did not develop the disease.

## Results

### Phospholipidic profiling

**Figure 1:**
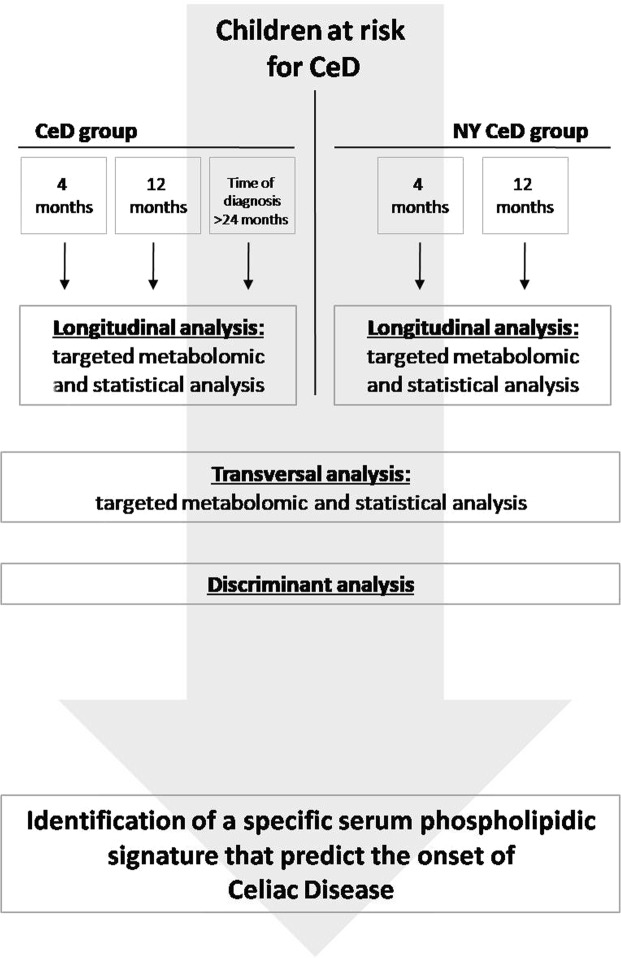
Study design: Phospholipid signature were investigated in the CeD and NY-CeD groups in longitudinal and transversal ways. In longitudinal analysis, the groups were compared according to their age: 4 months, 12 months and diagnosis time (>24 months). In transversal analysis, the groups were compared according to their diagnosis: CeD and NY-CeD. Discriminant analysis was performed to find a model that can predict the development of the disease.

**Figure 2:**
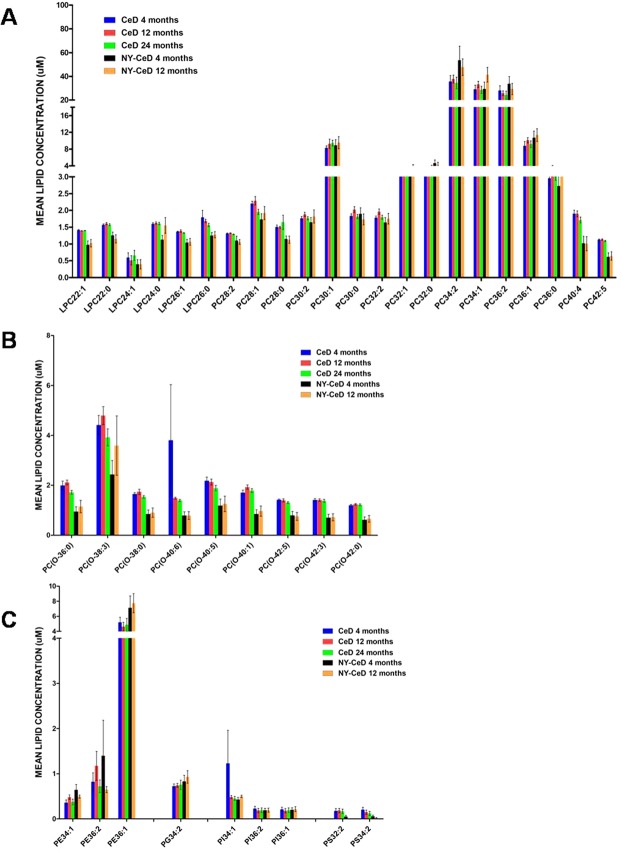
The mean lipid concentration in CeD at 4 and 12 months and at diagnosis time, and in NY-CeD at 4 and 12 months. Lyso-phosphatidylcholine, LPC, phosphatidylcholine, PC, (Panel A); alkylacyl-phosphatidylcholine, PC-O, (Panel B); phosphatidyletanolamine, PE, phosphatidylglycerol, PG, phosphatidylinositol, PI, phosphatidylserine, PS, (Panel C) are shown.

The serum phospholipid signature was measured in the CeD cases at 4 and 12 months and at diagnosis time (>24 months) and in the NY-CeD at 4 and 12 months (Fig. 1 and Supplemental Table 1S). A phospholipid dataset was obtained enrolling phospholipids present in more than 30% of the 327 measurements (Supplemental Table 2S). Figure 2 shows the mean values of each class for each time point in the CeD and NY-CeD.

No class of lipids showed significant differences between the ages of 4 and 12 months either in the CeD or in the NY-CeD. Only the PC36:0 showed a mean of 2,95 ± 0,22 µM at 4 months and 3,80 ± 0,26 µM at 12 months in the CeD, revealing a fold-change of 29% in CeD when compared with the control (F = 2,43; p = 0,019); however, it was not significant after the Bonferroni correction.

**Figure 3:**
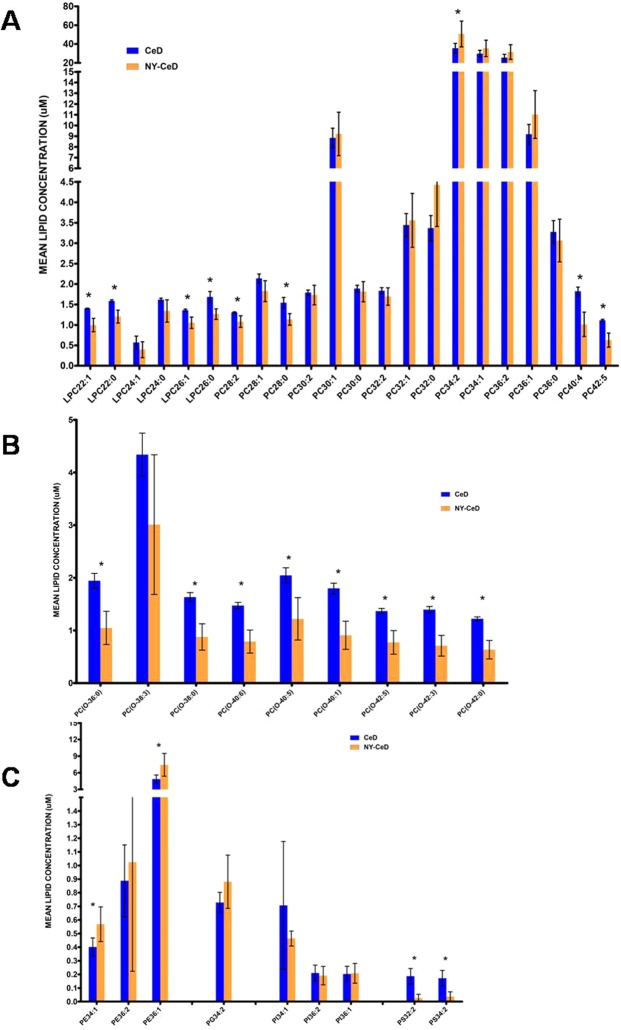
The mean lipid concentration in pooled times in CeD *versus* NY-CeD. Lyso-phosphatidylcholine, LPC, phosphatidylcholine, PC, (Panel A); alkylacyl-phosphatidylcholine, PC-O, (Panel B); phosphatidyletanolamine, PE, phosphatidylglycerol, PG, phosphatidylinositol, PI, phosphatidylserine, PS, (Panel C) are shown. (*) Significant differences between the mean values, Bonferroni corrected.

Given the homogeneity of the results, we pooled the lipid values across ages in order to increase the power of the study. Figure 3 shows the mean values of the lipids at pooled ages. Supplemental Table 3S reports the mean value concentration, replicates number and standard deviation; Supplemental Table 4S reports the difference of means with Bonferroni correction. The comparison between the two groups showed that lyso-phosphatidylcholine and phosphatidylcholine were significantly increased in the CeD *versus* NY-CeD (LPC22:1, LPC22:0, LPC26:1, LPC26:0; PC28:2, PC28:0, PC34:2, PC40:4 and PC42:5) (Fig. 3A). All classes of alkylacyl-phosphatidylcholine, from PC(O-36:0) to PC(O-42:0), show higher values in the CeD compared to NY-CeD (Fig. 3B). Phosphatidylethanolamines PE34:1 and PE36:1 were markedly lower in CeD *versus* NY-CeD and phosphatidylserines PS32:2 and PS34:2 were markedly higher in CeD versus NY-CeD (Fig. 3C).

**Figure 4:**
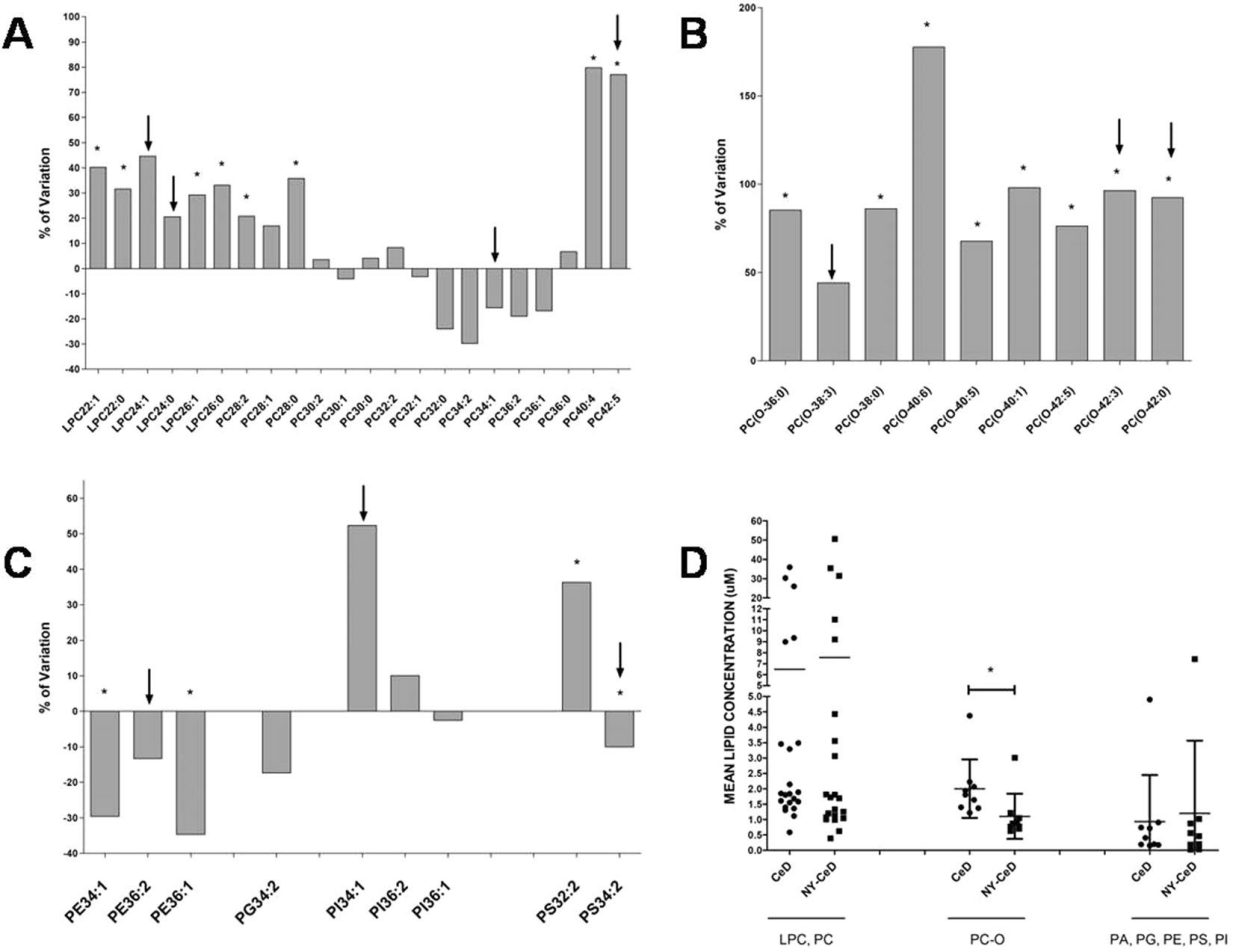
Fold change percentage of CeD versus NY-CeD. Lyso-phosphatidylcholine, LPC, phosphatidylcholine, PC, (Panel A); alkylacyl-phosphatidylcholine, PC-O, (Panel B); phosphatidyletanolamine, PE, phosphatidylglycerol, PG, phosphatidylinositol, PI, phosphatidylserine, PS, (Panel C) are shown. *Significant after Bonferroni correction. ↓Variable selected after discriminate analysis. The distribution of specific lipidic classes into two analysed groups CeD and NY-CeD are shown (Panel D)*.

To evaluate the size of the differences between CeD and NY-CeD, we computed the percentage change of CeD over NY-CeD expressed as: {[(mean values of CeD − mean values of NY-CeD) × 100]/mean values of NY-CeD} (Fig. 4). Phospholipids LPC22:1, LPC22:0, LPC26:1, LPC26:0, PC28:2, PC28:0, PC40:4, and PC42:5 were significantly overexpressed in CeD versus NY-CeD at average values of plus 40%, 31%, 29%, 32%, 21%, 36%, 81% and 77%, respectively (Fig. 4A). All alkylacyl-phosphatidylcholines, except PC(O-38:3), were significantly overexpressed in CeD versus NY-CeD: PC(O-36:0) 85%, PC(O-38:0) 87%, PC(O-40:6) 183%, PC(O-40:5) 69%, PC(O-40:1) 99%, PC(O-42:5) 77%, PC(O-42:3) 97%, and PC(O-42:0) 92% (Fig. 4B). Conversely, phosphatidylethanolamines PE34:1 and PE36:1 and phosphatidylserine PS34:2 appear significantly lower in CeD versus NY-CeD at −29%, −34%, and −12%, whereas PS32:2 was overexpressed of 36%.(Fig. 4C). The distribution of specific lipidic classes into the two analysed groups, CeD and NY-CeD, are reported in Fig. 4D.

The lipid serum values were compared in CeD and NY-CeD groups also in infants at 4 and 12 months of age, when gluten was not introduced in both cohorts (Supplemental Figs 1S and 2S). The differences observed do not seem to be related to the diet of children, in fact they are already evident at 4 months, when they do not yet eat gluten, are not related to the type of breastfeeding and do not change at 12 months, when children are on a free diet.

### Discriminant analysis in the lipidomic data set

To identify a profile able to better distinguish CeD from NY-CeD, we performed a multivariate stepwise discriminant analysis by two steps: the first with all classes of lipids, and the second with the classes of lipids that had no null values in any group. Each class of lipids was compared between the two groups and the lipid with the highest variance ratio (F) was first introduced into the model. Then, iteratively, all of the other classes of lipids were evaluated considered the variance explained by the previous class of lipids, up to the point that no other class of lipids contributed significantly to the model. Wilk’s lambda estimates the cumulative capacity to discriminate between the two groups once each best discriminating variable is added.

Since the changes within each group over time were mostly trivial (Fig. 2), serum phospholipid values at different time points were pooled to increase analytical power and accuracy. the results of the discriminant analysis were summerized in the Tables 1, 2, Table 1 shows the selected variables, and Table 2 the results of the unbiased classification. The model selects 11 variables: breast-feeding and PC(O-42:0), PC(O-38:3), PS34:2, LPC24:1, PE36:2, PC42:5, PC34:1, PI34:1, PC(O-42:3), and LPC24:0 lipids as the best discriminator parameters (Table 1). By this model, 61/66 CeD (92.5%) and 29/36 NY-CeD (80.5%) were correctly classified for an overall classification rate of 88.2%. To avoid self-assessment bias, a jack-knife auto-exclusion method was applied, obtaining a final correct classification rate of 85% (Table 2).

**Table 1:** Selected variables at pooled times.

Step	Variables	Wilks Lamba	Variance Ratio F	p
1	PC(O-42:0)	0,555	80,263	0
2	PC(O-38:3)	0,49	51,588	0
3	BREAST-FEADING	0,468	37,198	0
4	PS34:2	0,447	30,052	0
5	LPC24:1	0,43	25,424	0
6	PE36:2	0,419	21,959	0
7	PC42:5	0,408	19,518	0
8	PC34:1	0,397	17,683	0
9	PI34:1	0,387	16,221	0
10	PC(O-42:3)	0,375	15,195	0
11	LPC24:0	0,367	14,085	0

**Table 2:** Discriminat analysis at pooled times.

			Predicted Group		Total
			CeD	NY-CeD	
	Original Group	CeD	61 (92,5%)	5 (7,5%)	66
		NY-CeD	7 (19,5%)	29 (80,5%)	36
Cross-validated	Original Group	CeD	61 (92,5%)	5 (7,5%)	66
		NY-CeD	10 (28%)	26 (72%)	36

88,2% of individuals correctly classified.

85,0% of cross-validated individuals correctly classified.

Discriminant analysis at 4 months, before gluten introduction, selected 10 variables, breast-feeding and PC(O-42:3), PS32:2, PC(O-38:3), LPC26:0, PE36:2, PC28:2, LPC24:1, PC36:2, and PC30:1 lipids as the best discriminator parameters (Table 3). Since most of the lipid classes are inter correlated within the class (they follow a similar metabolic pathway), it was no surprise to observe some differences between the analysis at pooled ages and at 4 months only. The overall pathway is very similar indeed. Discriminant analysis (Table 4) at 4 months correctly classified 19/21 CeD (90.5%) and 17/18 NY-CeD (94.4%), for a total classification rate of 92.3%. A jack-knife auto-exclusion method was applied to correct the self-assessment bias of the previous discriminant analysis, obtaining a final classification rate of 82.1%.

**Table 3:** Selected variables at 4 months.

Step	Variables	Wilks Lamba	Variance Ratio F	p
1	PC(O-42:3)	0,56	29,014	0
2	BREAST-FEADING	0,531	15,893	0
3	PS32:2	0,496	11,833	0
4	PC(O-38:3)	0,46	9,975	0
5	LPC26:0	0,431	8,709	0
6	PE36:2	0,4	7,995	0
7	PC28:2	0,371	7,508	0
8	LPC24:1	0,349	6,996	0
9	PC36:2	0,324	6,721	0
10	PC30:1	0,299	6,572	0

**Table 4:** Discriminat analysis at 4 months.

			Predicted Group		Total
			CeD	NY-CeD	
	Original Group	CeD	19 (90,5%)	2 (9,5%)	21
		NY-CeD	1 (5,6%)	17 (94,4%)	18
Cross-validated	Original Group	CeD	19 (90,5%)	2 (9,5%)	21
		NY-CeD	5 (27,8%)	13 (72,2%)	18

92,3% of individuals correctly classified.

82,1% of cross-validated individuals correctly classified.

To explore a more robust discriminating model, we compared the serum lipid values of both groups at the ages of 4 and 12 months, excluding the values after 12 months that were available only for the CeD group. We also selected phospholipidic classes that had no null values in any of the children. Tables 5, 6 show the variables selected and the results of the unbiased classification at 4–12 months. A quite small set of lipids, together with the duration of breast-feeding, allowed for the development of a model that correctly predicted 88,2% of individuals: not one of the celiac patients was misclassified as NY-CeD, while 1/3^rd^ of the NY-CeD were misclassified as celiac patients. To correct for the over enthusiastic classification rate produced by classifying individuals with the same equation to which they contribute, an auto-exclusion jack-knife method was applied to recalculate an unbiased classification rate. As shown in Tables 7, 8 the best discriminator variables were constant and again none of the celiac cases were misclassified, while 1/3^rd^ of NY-CeD were allocated to the CeD group, for a total unbiased correct classification of 87,2% of individuals. Notably two alkylacyl-phospholipids (PC(O-42:0) and PC(O-38:3), together with one phosphatidylcholine (PC34:1) and breast-feeding, produced a specific signature of future progression to celiac disease.

**Table 5:** Selected variables at 4 and 12 months (no null values).

Step	Variables	WILKS′	Variance Ratio F	p
1	PC(O-42:0)	0,555	80,263	0
2	PC(O-38:3)	0,49	51,588	0
3	BREAST-FEADING	0,468	37,198	0
4	PC34:1	0,452	29,394	0

**Table 6:** Discriminat analysis at 4 and 12 months (no null values).

			Predicted Group		Total
			CeD	NY-CeD	
	Original Group	CeD	66 (100%)	0	66
		NY-CeD	12(33,3%)	24(66,7%)	36
Cross-validated	Original Group	CeD	66(100%)	0	66
		NY-CeD	13(36,1%)	23(64%)	36

88,2% of individuals correctly classified.

87,3% of cross-validated individuals correctly classified.

**Table 7:** Selected variables at 4 and 12 months (no null values).

Step	Insert Variables	Wilks Lamba	Variance Ratio F	p
1	PC(O-42:3)	0,56	29,014	0
2	BREAST-FEADING	0,531	15,893	0
3	PC(O-38:3)	0,503	11,516	0
4	PC34:1	0,474	9,431	0

Auto-exclusion jackknife method.

**Table 8:** Discriminat analysis at 4 and 12 months (no null values) Auto-exclusion jackknife method.

			Predicted Group		Total
			CeD	NY-CeD	
Original Group		CeD	20 (95,2%)	1 (4,8%)	21
		NY-CeD	4 (22,2%)	14(77,8)	18

87,2% of individuals correctly classified.

**Figure 5:**
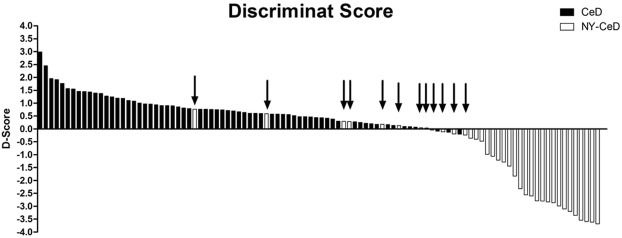
D-Score for each individual in our cohort. A positive D-score >0,273 corresponded to 80% probability to be in the CeD group on the left side of the graph, while a D-score <0,599 allocated 80% of cases to the NY-CeD group on the right side of the graph. The 5 CeD and 7 NY-CeD incorrectly classified are marked by arrows.

By the discriminant function developed in this analysis, a D-Score was calculated for each individual in our cohort. Each D-Score is associated with a probability of being classified into the CeD or NY-CeD group. Figure 5 shows the distribution of the D-Score of all individuals. CeDs have a positive score on the left, and NY-CeDs have a negative one: positive scores greater than 0,273 are associated with an 80%, or higher probability of being in the CeD group, while negative scores lower than −0,599 are associated with a probability of 80%, or higher, of being in the NY-CeD group. The individuals incorrectly classified are marked by arrows. It is easy to understand that individuals who have a D-Score from −0,3 to +0,3 could be easily misclassified.

However, the NY-CeD that showed a D-Score higher than 0,273 have a quite high risk to develop the disease in future. These cases have to be monitored in the near future, since we have screened them only up to 8 years to date, and they may still develop the disease in the future.

## Discussion

A large number of complex lipids have not only structural functions in the composition of membranes and organelles, but they have crucial signalling functions, over and above their nutritional function. Lipid metabolism is a hallmark of energy homeostasis, membrane structure, dynamic and signalling processes. Imbalances in lipid metabolism can therefore contribute to defining differential physiological phenotypes and pathological states, such as acute and chronic inflammatory condition^3^. Most lipids are synthetized in the endoplasmic reticulum, where their metabolism is continuously flowing with a cross-talk with vesicles and cell membranes^15^; the serum is likely to mirror the individual peculiar intracellular lipid metabolism.

In the present work, the phospholipid signature of a cohort of infants from families with one celiac proband and bearing the specific celiac HLA haplotype (DQ2 or DQ8) has been explored. A total of 33 infants developed CeD within 8 years of life while the rest of the cohort did not develop the disease up to 8 years of age. The genetic profiles between the groups may be somewhat similar for HLA, but the outcomes were very different. It is clear that genetics may not fully explain the differences found between the two groups. The HLA haplotype accounts for only approximately 40% of the genetic variance, while the 54 gene polymorphisms associated with CeD add no more than 10% to this level. To clarify the 50% ‘missing heredity’, we explored some epigenetic mechanisms, such as gene methylation^16^, microRNA^17^, and gene expression^18^, which are putatively involved in the CeD pathogenesis. These studies did not completely answer open questions. Since metabolomics is a complementary approach to study multifactorial disease, here, a targeted lipidomic platform has been used to investigate celiac disorders. LPC, PC, PC-O, PA, PE, PG, PI, and PS were monitored and quantified by a lipidomic platform, based on liquid chromatography coupled with mass spectrometry. Among PCs, we have measured monoacyl PC (lysoPC or LPC), di-acyl PC (PC) and alkylacyl-phosphatidylcholine (PC-O). Independent MRM methods have been employed to identify and quantify 6 LPC, 16PC, 9 PC-O, 11 PA, 11 PE, 4 PG, 9 PI, and 7 PS. The serum of 30 patients who became CeD was sampled at 3 time points (4 months, 12 months of life, and at the time of diagnosis >24 months), and the serum of 20 children who did not become CeD was sampled at 4 months and 12 months. These individuals were selected from a familial cohort and have been characterized according to their fatty acid profile to define a lipidomic signature that may predict the onset of celiac disease.

Surprisingly, the lipid profile appears to be fairly constant in each individual across the ages of 4, 12 and 24 months, both in the CeD and in the NY-CeD participants. This finding supports the hypothesis that the observed lipid variations are constitutive, since they are constant over time and already present at 4 months of age. This finding is in agreement with the recent work from Oresic group^12^. This ismight also be related to the small age interval of our cohort, since previous results showed an age effect only after 12 months of age on the serum phospholipid profile^10^.

The univariate analysis of the expression of each singular lipid may not provide an accurate picture of eventual differences between groups. A discriminant analysis selected a small lipid dataset able to correctly classify over 80% of subjects by 4 months, before exposure to gluten. The progression to celiac disease appears to be associated with a specific lipidomic signature characterized by reduction in some classes of phospholipids and compensative excesses of other classes.

In particular, we observed a peculiar distribution of the serum levels of mono- and diacyl-phosphatidylcholine classes. The serum LPC22:1, LPC22:0, LPC26:1, LPC26:0, PC28:2, PC28:0, PC40:4, and PC42:5 were increased in the CeD group. In addition, the PC34:1 was selected in the discriminant model to accurately differentiate infants with CeD versus NY-CeD.

The phosphatidylcholines account for approximately 50% of total cellular phospholipids and are the most abundant phospholipid in mammalian membranes. Most of the PC in the plasma membrane are found within the outer leaflet and perform an important structural role contributing not only to membrane integrity but also to intracellular vesicles functions. Recent studies have shown that vesicles might be a crucial key in the handling of gluten peptides in celiac patients^18^.

PC(40:4) showed the highest difference between CeD and NY-CeD. Indeed, the PC(40:4) was increased by +80% in CeD when compared with NY-CeD. Previous studies^8^ also identified PC(40:4) as a variable with the highest contribution to the projection score (VIP), exploring the cord serum lipidome in infants who developed three to four islet autoantibodies and a final progression to type 1 diabetes. They identified a specific cord blood lipidomic profile in infants who developed diabetes, featuring a reduction in major choline-containing phospholipids^8,10,12^.

It is interesting to note, in CeD, there is an increased level of LPC (LPC22:1, LPC22:0, LPC26:1, LPC26:0), which is defined as reactive hydrolytic products of PC as a consequence of phospholipase A2 activity. These molecules seem to impair endothelial barrier integrity, allowing for major plasticity. In addition, they induce the production of oxidant species through the activation of the NADH/NADPH oxidase system and increased chemokine expression^19^.

LPCs are the main component of oxidized low-density lipoprotein, produced as a hydrolysis product of PC. Indeed, LPC regulates various intracellular signalling events triggered by oxidized low density lipoproteins, participating in several immunologic and inflammatory processes. In the progression to CeD, the whole family of LPC is over-expressed. Increased LPC plasma levels have been found in several inflammatory diseases^20^. Moreover, dysregulation of LPC was shown to precede the appearance of islet autoantibodies in the study about progression to diabetes^10^.

Concerning alkylacyl-phosphatidylcholines, the CeD phospholipid signature shows a significantly increased serum level of a group of other phospholipids of the same family, constituted by PC(O-36:0), PC(O-38:0), PC(O-40:6), PC(O-40:5), PC(O-40:1), PC(O-42:5), PC(O-42:3), and PC(O-42:0)in CeD compared to NY-CeD. In particular, PC(O-34:2), PC(O-36:2), PC(O36:4), PC(O38:4), and PC(O40:4) showed high VIP scores in a study about diabetes progression^8,21^, a very significant and strong confirmation of their role in the development of an autoimmune disorder. The present Discriminant Analysis identifies just two members of this class, PC(O-42:0) and PC(O-38:3) that, together with breast-feeding and PC34:1, are capable of precisely identifying infants who will become celiac from those who will not, at 4 months of age, long before the appearance of the disease. They are ether-phospholipids, characterized by the presence of an ether bond. A great interest in these lipids arose because of the presence of a distinctive ether linkage, which limits the branching required for a structural function in membranes. Indeed, they are involved in specific intracellular signalling activities such as platelet-activating factor (PAF)^22,23^. PAF has been characterized as a new, ubiquitous, potent, and unique class of lipid chemical mediators that share similar biological activities, namely, PAF-like activity^22,23^. Under physiological conditions, the produced PAF participates in physiological processes such as reproduction, memory formation, vascular tone, apoptosis, and angiogenesis. However, in pathological conditions, excess amounts of PAF can cause inflammation and lead to inflammatory conditions or diseases, such as allergy, asthma, atherosclerosis, diabetes, renal diseases, cancer, HIV pathogenesis, and periodontitis^24–26^. Notably, several alkylacyl-phospholipids deficiencies are associated with peroxisomal disorders, membrane defects and cell viability.

Phosphatidylserine PS(32:2) was over represented in CeD infants. Due to their anionic serine head group, the PS localize in the inner face of plasmatic membrane, interacting to cationic proteins and promoting the assembly between proteins and their membrane receptors. Key cellular pathways, as apoptotic cellular phagocytosis and blood clotting, depend on membrane externalization of PS. Moreover, the PS improve the protein kinase C expression, affecting the cellular signalling mechanisms^27^. Finally, phosphatidylethanolamines PE(34:1) and PE(36:1) were decreased in CeD. PEs are localized in the membrane inner leaflet. Indeed, due to small dimension of their head group, the PEs were found to accomodate the protein insertions within the membrane. Their structural features allow PEs to promote formation of new vesicles and membrane fusion processes^27^. Our results reveal that the constant lipid profile characterizing the CeD group is evident long before (years) the CeD clinical manifestations or even the production of the specific anti-TG2 antibodies in very healthy and thriving infants at 4 months and is, thus, unlikely to be linked to an inflammation state. Indeed, we observed that the specific celiac lipid signature is present at 4 months of age and it remained constant over time, suggesting that it might be constitutive^6^.

We do acknowledge that the sizes of the samples are limited, being the precious results of an 8 years longitudinal study, but they are sufficient for the analysis by robust statistical methods; their strength is in the replication of the same findings at 4 and 12 months of age and the constant findings across multiple members of the same class of phospholipids. We are in the discovery phase and we do not pretend to propose this findings as diagnostic biomarkers for Celiac Disease, but as novel findings to elucidate the complex interplay between genetic predisposition, transcriptome and metabolome, before the introduction of gluten in the diet.

We have no clear explanation for why the lipid profile of infants who later develop CeD is so different from their peers with a similar genetic background that do not develop CeD, but these findings give further strong support that the ‘future CeD’ infants are born not only with a peculiar genomic background but also with a specific metabolomics profile, of which lipidomic profiling can provide a consistent view. As suggested by Oresic.^10–12^, this remarkable difference of serum phospholipids in infants who will go on to develop an autoimmune disease has to be explored in the domain of T-Cell immunity regulation and the series of epigenetic mechanisms able to drive genetically predisposed infants to their final outcome.

## Materials and Methods

### Subjects

From a cohort of 256 at risk-families for CeD^28^ we selected and analysed available serum samples from 30/33 infants who later became celiac (CeD) that were taken at 4 and 12 months of age and at the time of diagnosis of CeD, when patients were >24 months. Twenty infants from the same cohort who had not yet become celiac at 8 years of life (NY-CeD) were sampled at 4 and 12 months of age (Fig. 1). The description of the selected patients is summarized in Supplemental Table 1S. Identification code (ID), status, sex, time point analysed, time of diagnosis and breast-feeding duration were reported for each individual.

The study was carried out according to the Helsinki II Declaration and was approved by the Ethical Committee of the School of Medicine of the University of Naples Federico II, Protocol n. 191/06. The present research involving human participants under the age of 18 years (including donors of tissue samples). Each parent (and/or legal guardian) gave a fully informed consent to the participation of their child to the study and to the use of their biological samples for research purpose. The form is available on request at ‘r.auricchio@unina.it’.

The diagnosis of CeD was confirmed by a small intestinal biopsy following a twice positive test for serum anti-TG2 antibodies. The median age at diagnosis of CeD was 36.5 months (range 15–63 months). The mean duration of breast-feeding was 7.8 months in the CeD group and 5.5 months in the NY-CeD group with no significant differences. All infants were gradually introduced to gluten from the 6^th^ month of life, and none had received gluten at 4 months^28,29^. All blood samples (Table 1S), collected for this study, were centrifuged at 1300 × g, 20 min; +4 °C, 15 minutes after the blood collection; serum was frozen immediately at −80 °C, and stored until analysis.

### Lipidomic analysis

Analysis of lipid content was performed by ultraperformance liquid chromatography coupled to mass spectrometry^30,31^. Our lipidomic platform was set to detect different molecules belonging to LPC, PC, PC-O, PE, PG, PI and PS families^32,33^. Three technical replicates were carried out for each analysed sample. A standard mixture, containing heavy D-α-phosphatidylcholine, dipalmitoyl (d80, 98%), d6-L-α-phosphatidylethanolamine dioleoyl (Avanti Polar Lipids) was added to each human serum aliquot. Serum samples aliquots (100 µL) were treated according to Burè *et al*.^34–36^. The samples were dissolved in 990 μL CHCl_3_/CH_3_OH 17:1 (v/v) and centrifuged at 13000 rpm for 5 min. The supernatants containing apolar lipids was removed and the pellets were suspended in 990 μL of chloroform/MeOH 2:1 (v/v) to extract the polar lipids. They were then centrifuged for 10 min at 13000 rpm. The supernatant was collected and stored at −20 °C until the subsequent mass spectrometric analysis. The samples were analysed by using an AB Sciex QTrap 4000 mass spectrometer, coupled with an ExpressHT™-Ultra HPLC system (Eksigent)^33,34^. An aliquot of 5 µL of the extract was injected and separated on a Halo C18 1.0 mm × 50 mm, 2.7 µm column using a 40 μL/min flow rate at 40 °C with a 12 min gradient (0 min 70% A, 2 min 50% A, 9 min 5% A, 11 min 50% A, 12 min 70% A). The auto sampler was cooled at 4 °C. Solvent A composition: CH_3_CN/CH_3_OH/H2O 97:2:1 v/v/v +0.2% CH_3_COOH +5 mM CH_3_COONH_4_; solvent B composition: CH_3_OH +0.2% CH_3_COOH +5 mM CH_3_COONH_4_. The mass analysis was carried out in positive mode (ESI+) to detect LPC, PC, PC-O, and in negative mode (ESI-) to detect PE, PG, PI, PS. The measured phospholipidic classes concentrations are listed in Supplemental Table 2S. For each phospholipids compound, precursor ions, product ions and optimal collision energies were established for each MRM transition using the Analyst software^32,33^. The instrumental setting was: CUR 20, CAD 5, IS 4500, TEMP 380, GS1 25, GS2 24. The MS data were normalized according to the internal standard MS signal. The mass spectrometry procedure details of MRM/MS method used to detect PC (positive ione mode) and PA PI, PE, PG, PS (negative ione mode) are listed in Tables 5S and 6S, respectively.

### Statistical analysis

Phospholipids present in more than 30% of the 327 measurements were included in the dataset. The difference between mean values of each lipid in the two groups (CeD and NY-CeD) was evaluated by Student’s t test, with a Bonferroni correction for multiple comparison within each class of lipids. The percentage changes of CeD versus NY-CeD was computed as: [(mean of NY-CeDs − mean of CeDs) * 100)/mean of NY-CeDs]. A further analysis was carried out selecting phospholipids with no 0 values in any class. Principal component analysis, using XLStat 2018, of the pre-processed data was initially performed to highlight patterns and to try to identify clusters of lipids.

### Predictive models

A stepwise discriminant analysis was used to select variables able to differentiate children who became celiac (CeD) from those who did not became celiac by 8 years (NY-CeD). Wilk’s lambda, ranging from 1 (complete overlap) to 0 (complete distance) estimates the capacity of each selected variable to discriminate between the groups. By the Discriminant score (D-Score) obtained through the model, it is possible to classify (predict) the individuals into the CeD or NY-CeD group, using the selected variables, blinded to the final diagnosis. To avoid an overenthusiastic correct classification of cases, when the model obtained by the analysis is used to classify the same cases by which the model was obtained, we applied a jack-knife auto-exclusion method. Each case was classified after the exclusion of the same case from the development of the model, iteratively for all cases. A D-Score was calculated^37^ by multiplying the normalized value of each variable included in the stepwise discriminant equation to its respective regression coefficient. From the score, the individual probability to be assigned to one or the other group was derived. Statistical analyses were performed with SPSS 17.0 (SPSS Inc., Chicago, IL, USA).

## Supplementary information

Supplemental Table 1S

Supplemental Table 3S

Supplemental Table 4S

Supplemental Table 5S

Supplemental Table 6S

Supplemental Figures

Supplemental Table 2S
