# Supplementary material for: A Phospholipid Profile at 4 Months Predicts the Onset of Celiac Disease in at-Risk Infants
Source: Sci Rep. 2019 Oct 4;9:14303. doi: 10.1038/s41598-019-50735-7 (PMC6778072; doi:10.1038/s41598-019-50735-7)
Supplement: Supplementary file 1 — Supplemental Table 1S [file 41598_2019_50735_MOESM1_ESM.pdf]

A PHOSPHOLIPID PROFILE AT 4 MONTHS PREDICTS THE ONSET OF CELIAC DISEASE IN AT-RISK INFANTS

R. Auricchio[1,2], M. Galatola[1,2], D. Cielo[1,2], A. Amoresano[3], M. Caterino[4,5], E. De Vita[3], A. Illiano[3], R.Troncone[1,2], L. Greco[1,2]and M. Ruoppolo[4,5]

Table 1S: Individual characteristics of patients enrolled in the study

ID	Status	Sex	Time 4 months	Time 12 months	Time of diagnosis (Months)	Breastfeding (Months)
LIP01	CeD	F	Analyzed	Analyzed	20	1,5
LIP02	CeD	F	Analyzed	Analyzed	15	12
LIP03	CeD	M	Analyzed	Analyzed	34	7,5
LIP04	CeD	M	Analyzed	Analyzed	51	5
LIP05	CeD	F	Analyzed	Analyzed	37	4
LIP06	CeD	F	Analyzed	Analyzed	21	3
LIP07	CeD	F	Analyzed	Analyzed	34	2
LIP08	CeD	M	Analyzed	Analyzed	48	18
LIP09	CeD	F	Analyzed	Analyzed	27	0
LIP10	CeD	F	Analyzed	Analyzed	36	2
LIP11	CeD	M	Analyzed	Analyzed	20	4
LIP12	CeD	M	Analyzed	Analyzed	60	11
LIP13	CeD	F	Analyzed	Analyzed	51	12
LIP14	CeD	M	Analyzed	Analyzed	48	3
LIP15	CeD	F	Analyzed	Analyzed	63	6
LIP16	CeD	F	N.A.	Analyzed	27	2
LIP17	CeD	M	Analyzed	N.A.	40	24
LIP18	CeD	F	Analyzed	Analyzed	N.A.	15
LIP19	CeD	M	Analyzed	Analyzed	N.A.	18
LIP20	CeD	F	Analyzed	Analyzed	42	7
LIP21	CeD	F	Analyzed	N.A.	N.A.	2
LIP22	CeD	F	N.A.	N.A.	24	7
LIP23	CeD	F	Analyzed	N.A.	N.A.	N.A.
LIP24	CeD	M	N.A.	N.A.	26	5
LIP25	CeD	M	N.A.	N.A.	27	8
LIP26	CeD	M	N.A.	Analyzed	N.A.	15
LIP27	CeD	F	N.A.	Analyzed	43	8
LIP28	CeD	F	Analyzed	Analyzed	N.A.	6
LIP29	CeD	M	Analyzed	Analyzed	N.A.	12
LIP30	CeD	M	N.A.	Analyzed	46	N.A.
LIP31	NY-CeD	F	Analyzed	Analyzed	N.A.	4,5
LIP32	NY-CeD	M	Analyzed	Analyzed	N.A.	1
LIP33	NY-CeD	M	Analyzed	Analyzed	N.A.	1,5
LIP34	NY-CeD	M	Analyzed	Analyzed	N.A.	24
LIP35	NY-CeD	F	Analyzed	Analyzed	N.A.	4
LIP36	NY-CeD	F	Analyzed	Analyzed	N.A.	2
LIP37	NY-CeD	F	Analyzed	Analyzed	N.A.	0
LIP38	NY-CeD	F	Analyzed	Analyzed	N.A.	5
LIP39	NY-CeD	F	Analyzed	Analyzed	N.A.	0
LIP40	NY-CeD	F	Analyzed	Analyzed	N.A.	9
LIP41	NY-CeD	F	Analyzed	Analyzed	N.A.	0
LIP42	NY-CeD	F	Analyzed	Analyzed	N.A.	5
LIP43	NY-CeD	M	Analyzed	Analyzed	N.A.	18
LIP44	NY-CeD	M	Analyzed	Analyzed	N.A.	4

LIP45	NY-CeD	M	Analyzed	Analyzed	N.A.	0
LIP46	NY-CeD	F	Analyzed	Analyzed	N.A.	5
LIP47	NY-CeD	F	Analyzed	Analyzed	N.A.	5
LIP48	NY-CeD	M	Analyzed	Analyzed	N.A.	11
LIP49	NY-CeD	M	Analyzed	Analyzed	N.A.	6
LIP50	NY-CeD	M	Analyzed	Analyzed	N.A.	N.A.
