# Supplementary material for: A Phospholipid Profile at 4 Months Predicts the Onset of Celiac Disease in at-Risk Infants
Source: Sci Rep. 2019 Oct 4;9:14303. doi: 10.1038/s41598-019-50735-7 (PMC6778072; doi:10.1038/s41598-019-50735-7)
Supplement: Supplementary file 2 — Supplemental Table 3S [file 41598_2019_50735_MOESM2_ESM.pdf]

# A PHOSPHOLIPID PROFILE AT 4 MONTHS PREDICTS THE ONSET OF CELIAC DISEASE IN AT-RISK INFANTS

R. Auricchio[1,2], M. Galatola[1,2], D. Cielo[1,2], A. Amoresano[3], M. Caterino[4,5], E. De Vita[3], A. Illiano[3], R.Troncone[1,2], L. Greco[1,2]and M. Ruoppolo[4,5]

**Table 3S. Media phospholipidic concentrations**

| | CeD | | | NY- CeD | | |
|---|---|---|---|---|---|---|
| | Mean | N | SD | Mean | N | SD |
| **LPC22:1** | 1,39990 | 69 | ,044681 | ,99834 | 40 | ,511025 |
| **LPC24:1** | ,56974 | 69 | ,653240 | ,39378 | 40 | ,607639 |
| **LPC24:0** | 1,61618 | 69 | ,160061 | 1,34050 | 40 | ,852915 |
| **LPC26:1** | 1,35986 | 69 | ,123126 | 1,05254 | 40 | ,435883 |
| **LPC26:0** | 1,68181 | 69 | ,576134 | 1,26368 | 40 | ,405089 |
| **PC28:2** | 1,30676 | 69 | ,056811 | 1,08224 | 40 | ,445423 |
| **PC28:0** | 1,54280 | 69 | ,532949 | 1,13608 | 40 | ,442318 |
| **PC30:2** | 1,79275 | 69 | ,268079 | 1,73182 | 40 | ,752678 |
| **PC30:1** | 8,82783 | 69 | 3,828063 | 9,20317 | 40 | 6,334366 |
| **PC30:0** | 1,88696 | 69 | ,348999 | 1,81283 | 40 | ,773155 |
| **PC32:2** | 1,83546 | 69 | ,317367 | 1,69513 | 40 | ,671073 |
| **PC32:1** | 3,44354 | 69 | 1,172975 | 3,55780 | 40 | 2,063065 |
| **PC32:0** | 3,36604 | 69 | 1,288380 | 4,43028 | 40 | 3,194342 |
| **PC34:2** | 35,62965 | 69 | 20,905974 | 50,70896 | 40 | 42,736552 |
| **PC34:1** | 29,85559 | 69 | 14,502616 | 35,39594 | 40 | 27,097492 |
| **PC36:2** | 25,55327 | 69 | 15,236386 | 31,51625 | 40 | 24,107880 |
| **PC36:1** | 9,16744 | 69 | 3,893604 | 11,02008 | 40 | 6,959444 |
| **PC36:0** | 3,27367 | 69 | 1,166584 | 3,06698 | 40 | 1,632921 |
| **PC40:4** | 1,82213 | 69 | ,434496 | 1,01382 | 40 | ,927552 |
| **PC42:5** | 1,11407 | 69 | ,086906 | ,62913 | 40 | ,543191 |
| **PC(O-36:0)** | 1,94324 | 69 | ,585253 | 1,04807 | 40 | ,987178 |
| **PC(O-38:0)** | 1,63179 | 69 | ,363827 | ,87657 | 40 | ,783217 |
| **PC(O-40:6)** | 2,18879 | 69 | 5,925085 | ,78807 | 40 | ,684057 |
| **PC(O-40:5)** | 2,04606 | 69 | ,594349 | 1,21982 | 40 | 1,259169 |
| **PC(O-40:1)** | 1,79749 | 69 | ,422537 | ,90765 | 40 | ,837874 |
| **PC(O-42:5)** | 1,36449 | 69 | ,222076 | ,77398 | 40 | ,701620 |
| **PC(O-42:3)** | 1,39406 | 69 | ,250391 | ,70990 | 40 | ,617467 |
| **PC(O-42:0)** | 1,22053 | 69 | ,165540 | ,63420 | 40 | ,548703 |
| **PE34:1** | ,40088 | 69 | ,275728 | ,56908 | 40 | ,397639 |
| **PE36:1** | 4,85536 | 69 | 3,138639 | 7,43028 | 40 | 6,384459 |
| **PG34:2** | ,72784 | 69 | ,313127 | ,88043 | 40 | ,609955 |
| **PI34:1** | ,70643 | 69 | 1,954880 | ,46374 | 40 | ,172897 |
| **PI36:2** | ,21023 | 69 | ,241520 | ,19103 | 40 | ,211858 |
| **PI36:1** | ,20322 | 69 | ,233099 | ,20842 | 40 | ,227062 |
| **PS32:2** | ,18724 | 69 | ,234548 | ,02601 | 40 | ,089292 |
| **PS34:2** | ,17220 | 69 | ,236671 | ,03715 | 40 | ,110329 |
