# Supplementary material for: A Phospholipid Profile at 4 Months Predicts the Onset of Celiac Disease in at-Risk Infants
Source: Sci Rep. 2019 Oct 4;9:14303. doi: 10.1038/s41598-019-50735-7 (PMC6778072; doi:10.1038/s41598-019-50735-7)
Supplement: Supplementary file 3 — Supplemental Table 4S [file 41598_2019_50735_MOESM3_ESM.pdf]

A PHOSPHOLIPID PROFILE AT 4 MONTHS PREDICTS THE ONSET OF CELIAC DISEASE IN AT-RISK INFANTS

R. Auricchio[1,2], M. Galatola[1,2], D. Cielo[1,2], A. Amoresano[3], M. Caterino[4,5], E. De Vita[3], A. Illiano[3], R.Troncone[1,2], L. Greco[1,2]and M. Ruoppolo[4,5]

Table 4S. Statistic parameters

		df	Mean Square	F	Sig.
LPC22:1	Between Groups	1	4,083	42,332	**,000**
	Within Groups	107	,096		
LPC22:0	Between Groups	1	3,656	37,629	**,000**
	Within Groups	107	,097		
LPC24:1	Between Groups	1	,784	1,932	,167
	Within Groups	107	,406		
LPC24:0	Between Groups	1	1,924	6,838	,010
	Within Groups	107	,281		
LPC26:1	Between Groups	1	2,391	30,315	**,000**
	Within Groups	107	,079		
LPC26:0	Between Groups	1	4,427	16,350	**,000**
	Within Groups	107	,271		
PC28:2	Between Groups	1	1,276	17,164	**,000**
	Within Groups	107	,074		
PC28:1	Between Groups	1	2,425	6,516	,012
	Within Groups	107	,372		
PC28:0	Between Groups	1	4,189	16,634	**,000**
	Within Groups	107	,252		
PC30:2	Between Groups	1	,094	,373	,543
	Within Groups	107	,252		
PC30:1	Between Groups	1	3,567	,149	,700
	Within Groups	107	23,938		
PC30:0	Between Groups	1	,139	,471	,494
	Within Groups	107	,295		
PC32:2	Between Groups	1	,499	2,185	,142
	Within Groups	107	,228		
PC32:1	Between Groups	1	,331	,136	,713
	Within Groups	107	2,426		
PC32:0	Between Groups	1	28,679	6,007	,016
	Within Groups	107	4,774		
PC34:2	Between Groups	1	5757,654	6,103	,015
	Within Groups	107	943,459		
PC34:1	Between Groups	1	777,244	1,937	,167
	Within Groups	107	401,298		
PC36:2	Between Groups	1	900,346	2,505	,116
	Within Groups	107	359,369		
PC36:1	Between Groups	1	86,909	3,185	,077
	Within Groups	107	27,288		
PC36:0	Between Groups	1	1,082	,589	,445
	Within Groups	107	1,837		
PC40:4	Between Groups	1	16,544	38,158	**,000**
	Within Groups	107	,434		
PC42:5	Between Groups	1	5,955	53,003	**,000**
	Within Groups	107	,112		

PC(O-36:0)	Between Groups	1	20,291	35,419	,000
	Within Groups	107	,573		
PC(O-38:3)	Between Groups	1	44,712	5,503	,021
	Within Groups	107	8,125		
PC(O-38:0)	Between Groups	1	14,442	46,934	,000
	Within Groups	107	,308		
PC(O-40:6)	Between Groups	1	49,681	2,210	,140
	Within Groups	107	22,481		
PC(O-40:5)	Between Groups	1	17,286	21,543	,000
	Within Groups	107	,802		
PC(O-40:1)	Between Groups	1	20,050	54,284	,000
	Within Groups	107	,369		
PC(O-42:5)	Between Groups	1	8,830	41,892	,000
	Within Groups	107	,211		
PC(O-42:3)	Between Groups	1	11,852	66,283	,000
	Within Groups	107	,179		
PC(O-42:0)	Between Groups	1	8,705	68,461	,000
	Within Groups	107	,127		
PE34:1	Between Groups	1	,716	6,761	,011
	Within Groups	107	,106		
PE36:2	Between Groups	1	,471	,155	,695
	Within Groups	107	3,043		
PE36:1	Between Groups	1	167,883	7,950	,006
	Within Groups	107	21,117		
PG34:2	Between Groups	1	,590	2,979	,087
	Within Groups	107	,198		
PI34:1	Between Groups	1	1,491	,611	,436
	Within Groups	107	2,440		
PI36:2	Between Groups	1	,009	,175	,677
	Within Groups	107	,053		
PI36:1	Between Groups	1	,001	,013	,910
	Within Groups	107	,053		
PS32:2	Between Groups	1	,658	17,382	,000
	Within Groups	107	,038		
PS34:2	Between Groups	1	,462	11,535	,001
	Within Groups	107	,040		
