# Supplementary material for: A Phospholipid Profile at 4 Months Predicts the Onset of Celiac Disease in at-Risk Infants
Source: Sci Rep. 2019 Oct 4;9:14303. doi: 10.1038/s41598-019-50735-7 (PMC6778072; doi:10.1038/s41598-019-50735-7)
Supplement: Supplementary file 4 — Supplemental Table 5S [file 41598_2019_50735_MOESM4_ESM.pdf]

A PHOSPHOLIPID PROFILE AT 4 MONTHS PREDICTS THE ONSET OF CELIAC DISEASE IN AT-RISK INFANTS

R. Auricchio[1,2], M. Galatola[1,2], D. Cielo[1,2], A. Amoresano[3], M. Caterino[4,5], E. De Vita[3], A. Illiano[3], R.Troncone[1,2], L. Greco[1,2]and M. Ruoppolo[4,5]

Table 5S: MRM/MS method in positive ione mode for PC

ID	Q1 (m/z)	Q3 (m/z)	MODE	DP (V)	CE (V)	DWT (msec)	CXP (V)
**D80-PC**	814.53	814.53	POS	40	47	25	9
**LPC22:1**	592,4	184,1	POS	40	47	25	9
**LPC22:0**	594,4	184,1	POS	40	47	25	9
**LPC24:1**	620,4	184,1	POS	40	47	25	9
**LPC24:0**	622,4	184,1	POS	40	47	25	9
**LPC26:1**	648,5	184,1	POS	40	47	25	9
**LPC26:0**	650,5	184,1	POS	40	47	25	9
**PC28:2**	674,5	184,1	POS	40	47	25	9
**PC28:1**	676,5	184,1	POS	40	47	25	9
**PC28:0**	678,5	184,1	POS	40	47	25	9
**PC30:2**	702,5	184,1	POS	40	47	25	9
**PC30:1**	704,5	184,1	POS	40	47	25	9
**PC30:0**	706,5	184,1	POS	40	47	25	9
**PC32:2**	730,5	184,1	POS	40	47	25	9
**PC32:1**	732,6	184,1	POS	40	47	25	9
**PC32:0**	734,6	184,1	POS	40	47	25	9
**PC34:2**	758,6	184,1	POS	40	47	25	9
**PC34:1**	760,6	184,1	POS	40	47	25	9
**PC36:2**	786,6	184,1	POS	40	47	25	9
**PC36:1**	788,6	184,1	POS	40	47	25	9
**PC36:0**	790,6	184,1	POS	40	47	25	9
**PC40:4**	838,6	184,1	POS	40	47	25	9
**PC42:5**	850,6	184,1	POS	40	47	25	9
**PC(O-36:0)**	776,6	184,1	POS	40	47	25	9
**PC(O-38:3)**	798,6	184,1	POS	40	47	25	9
**PC(O-38:0)**	804,6	184,1	POS	40	47	25	9
**PC(O-40:6)**	820,6	184,1	POS	40	47	25	9
**PC(O-40:5)**	822,6	184,1	POS	40	47	25	9
**PC(O-40:1)**	830,7	184,1	POS	40	47	25	9
**PC(O-42:5)**	864,6	184,1	POS	40	47	25	9
**PC(O-42:3)**	854,6	184,1	POS	40	47	25	9
**PC(O-42:0)**	860,7	184,1	POS	40	47	25	9
