# Supplementary material for: A Phospholipid Profile at 4 Months Predicts the Onset of Celiac Disease in at-Risk Infants
Source: Sci Rep. 2019 Oct 4;9:14303. doi: 10.1038/s41598-019-50735-7 (PMC6778072; doi:10.1038/s41598-019-50735-7)
Supplement: Supplementary file 5 — Supplemental Table 6S [file 41598_2019_50735_MOESM5_ESM.pdf]

# A PHOSPHOLIPID PROFILE AT 4 MONTHS PREDICTS THE ONSET OF CELIAC DISEASE IN AT-RISK INFANTS

R. Auricchio[1,2], M. Galatola[1,2], D. Cielo[1,2], A. Amoresano[3], M. Caterino[4,5], E. De Vita[3], A. Illiano[3], R.Troncone[1,2], L. Greco[1,2]and M. Ruoppolo[4,5]

**Table 6S:** MRM/MS method in negative ione mode for PA, PI, PE, PG, PS.

| ID | Q1 (m/z) | Q3 (m/z) | MODE | DP (V) | CE (V) | DWT (msec) | CXP (V) |
|---|---|---|---|---|---|---|---|
| **PA 28:1** | 597,4 | 171,1 | NEG | -180 | -50 | 25 | -13 |
| | | 199,2 | NEG | -180 | -50 | 25 | -13 |
| | | 253,2 | NEG | -180 | -50 | 25 | -13 |
| | | 281,2 | NEG | -180 | -50 | 25 | -13 |
| **PA 28:0** | 590,4 | 171,1 | NEG | -180 | -50 | 25 | -13 |
| | | 199,2 | NEG | -180 | -50 | 25 | -13 |
| | | 255,2 | NEG | -180 | -50 | 25 | -13 |
| | | 283,2 | NEG | -180 | -50 | 25 | -13 |
| **PA 30:1** | 616,4 | 227,2 | NEG | -180 | -50 | 25 | -13 |
| | | 253,2 | NEG | -180 | -50 | 25 | -13 |
| **PA 30:0** | 618,4 | 199,2 | NEG | -180 | -50 | 25 | -13 |
| | | 227,2 | NEG | -180 | -50 | 25 | -13 |
| | | 255,2 | NEG | -180 | -50 | 25 | -13 |
| | | 283,2 | NEG | -180 | -50 | 25 | -13 |
| **PA 32:2** | 642,4 | 253,2 | NEG | -180 | -50 | 25 | -13 |
| **PA 32:1** | 644,4 | 227,2 | NEG | -180 | -50 | 25 | -13 |
| | | 253,2 | NEG | -180 | -50 | 25 | -13 |
| | | 281,3 | NEG | -180 | -50 | 25 | -13 |
| **PA 32:0** | 646,4 | 255,2 | NEG | -180 | -50 | 25 | -13 |
| **PA 34:2** | 670,5 | 253,2 | NEG | -180 | -50 | 25 | -13 |
| | | 281,3 | NEG | -180 | -50 | 25 | -13 |
| **PA 34:1** | 672,5 | 253,2 | NEG | -180 | -50 | 25 | -13 |
| | | 283,3 | NEG | -180 | -50 | 25 | -13 |
| | | 281,3 | NEG | -180 | -50 | 25 | -13 |
| | | 255,2 | NEG | -180 | -50 | 25 | -13 |
| **PA 36:2** | 698,5 | 281,3 | NEG | -180 | -50 | 25 | -13 |
| **PA 36:1** | 700,5 | 281,3 | NEG | -180 | -50 | 25 | -13 |
| | | 283,3 | NEG | -180 | -50 | 25 | -13 |
| **D6-PE** | 750.04 | 750.04 | NEG | -160 | -48 | 25 | -11 |
| **PE 28:1** | 631,4 | 199,2 | NEG | -160 | -48 | 25 | -11 |
| | | 253,2 | NEG | -160 | -48 | 25 | -11 |
| **PE 28:0** | 633,4 | 199,2 | NEG | -160 | -48 | 25 | -11 |
| | | 255,5 | NEG | -160 | -48 | 25 | -11 |
| **PE 30:2** | 657,5 | 225,2 | NEG | -160 | -48 | 25 | -11 |
| | | 253,2 | NEG | -160 | -48 | 25 | -11 |
| **PE 30:1** | 659,5 | 199,2 | NEG | -160 | -48 | 25 | -11 |
| | | 227,2 | NEG | -160 | -48 | 25 | -11 |
| | | 253,2 | NEG | -160 | -48 | 25 | -11 |
| | | 281,3 | NEG | -160 | -48 | 25 | -11 |
| **PE 30:0** | 661,5 | 199,2 | NEG | -160 | -48 | 25 | -11 |
| | | 227,2 | NEG | -160 | -48 | 25 | -11 |

| | | | | | | | |
|---|---|---|---|---|---|---|---|
| | | 255,2 | NEG | -160 | -48 | 25 | -11 |
| | | 283,2 | NEG | -160 | -48 | 25 | -11 |
| **PE 32:2** | 685,5 | 253,3 | NEG | -160 | -48 | 25 | -11 |
| | | 227,2 | NEG | -160 | -48 | 25 | -11 |
| **PE 32:1** | 687,5 | 281,3 | NEG | -160 | -48 | 25 | -11 |
| | | 255,2 | NEG | -160 | -48 | 25 | -11 |
| | | 253,2 | NEG | -160 | -48 | 25 | -11 |
| **PE 34:2** | 713,5 | 253,2 | NEG | -160 | -48 | 25 | -11 |
| | | 281,3 | NEG | -160 | -48 | 25 | -11 |
| **PE 34:1** | 715,5 | 255,2 | NEG | -160 | -48 | 25 | -11 |
| | | 281,3 | NEG | -160 | -48 | 25 | -11 |
| **PE 36:2** | 741,5 | 281,3 | NEG | -160 | -48 | 25 | -11 |
| **PE 36:1** | 743,5 | 281,3 | NEG | -160 | -48 | 25 | -11 |
| | | 283,3 | NEG | -160 | -48 | 25 | -11 |
| **PG 32:2** | 716,5 | 253,2 | NEG | -130 | -48 | 25 | -11 |
| **PG 32:1** | 718,5 | 253,2 | NEG | -130 | -48 | 25 | -11 |
| | | 255,2 | NEG | -130 | -48 | 25 | -11 |
| **PG 34:2** | 744,5 | 253,2 | NEG | -130 | -48 | 25 | -11 |
| | | 281,3 | NEG | -130 | -48 | 25 | -11 |
| **PG 34:1** | 746,6 | 255,2 | NEG | -130 | -48 | 25 | -11 |
| | | 281,3 | NEG | -130 | -48 | 25 | -11 |
| **PS 32:3** | 729,5 | 253,2 | NEG | -90 | -60 | 25 | -33 |
| | | 227,2 | NEG | -90 | -60 | 25 | -33 |
| **PS 32:1** | 731,5 | 253,2 | NEG | -90 | -60 | 25 | -33 |
| | | 255,2 | NEG | -90 | -60 | 25 | -33 |
| | | 281,3 | NEG | -90 | -60 | 25 | -33 |
| **PS 32:0** | 733,5 | 255,2 | NEG | -90 | -60 | 25 | -33 |
| | | 253,2 | NEG | -90 | -60 | 25 | -33 |
| **PS 34:2** | 757,6 | 281,3 | NEG | -90 | -60 | 25 | -33 |
| | | 255,2 | NEG | -90 | -60 | 25 | -33 |
| **PS 34:1** | 759,6 | 255,2 | NEG | -90 | -60 | 25 | -33 |
| | | 281,3 | NEG | -90 | -60 | 25 | -33 |
| **PS 32:2** | 785,6 | 281,3 | NEG | -90 | -60 | 25 | -33 |
| **PS 36:0** | 789,6 | 283,3 | NEG | -90 | -60 | 25 | -33 |
| **PI 26:1** | 722,5 | 171,1 | NEG | -85 | -62 | 25 | -10 |
| | | 253,2 | NEG | -85 | -62 | 25 | -10 |
| | | 171,1 | NEG | -85 | -62 | 25 | -10 |
| **PI 26:0** | 724,5 | 199,2 | NEG | -85 | -62 | 25 | -10 |
| | | 227,2 | NEG | -85 | -62 | 25 | -10 |
| | | 255,2 | NEG | -85 | -62 | 25 | -10 |
| | | 199,2 | NEG | -85 | -62 | 25 | -10 |
| **PI 28:1** | 750,6 | 225,2 | NEG | -85 | -62 | 25 | -10 |
| | | 227,2 | NEG | -85 | -62 | 25 | -10 |
| | | 253,2 | NEG | -85 | -62 | 25 | -10 |
| **PI 28:0** | 752,6 | 171,1 | NEG | -85 | -62 | 25 | -10 |
| | | 199,2 | NEG | -85 | -62 | 25 | -10 |

| | | | | | | | |
|---|---|---|---|---|---|---|---|
| | | 227,2 | NEG | -85 | -62 | 25 | -10 |
| | | 255,2 | NEG | -85 | -62 | 25 | -10 |
| | | 283,3 | NEG | -85 | -62 | 25 | -10 |
| **PI 30:2** | 776,6 | 253,2 | NEG | -85 | -62 | 25 | -10 |
| | | 225,2 | NEG | -85 | -62 | 25 | -10 |
| | | 199,2 | NEG | -85 | -62 | 25 | -10 |
| **PI 30:1** | 778,6 | 225,2 | NEG | -85 | -62 | 25 | -10 |
| | | 227,2 | NEG | -85 | -62 | 25 | -10 |
| | | 253,2 | NEG | -85 | -62 | 25 | -10 |
| | | 199,2 | NEG | -85 | -62 | 25 | -10 |
| **PI 30:0** | 780,6 | 227,2 | NEG | -85 | -62 | 25 | -10 |
| | | 255,2 | NEG | -85 | -62 | 25 | -10 |
| | | 283,3 | NEG | -85 | -62 | 25 | -10 |
| **PI 32:2** | 804,6 | 253,2 | NEG | -85 | -62 | 25 | -10 |
| | | 227,2 | NEG | -85 | -62 | 25 | -10 |
| **PI 32:1** | 806,6 | 225,2 | NEG | -85 | -62 | 25 | -10 |
| | | 283,3 | NEG | -85 | -62 | 25 | -10 |
| | | 253,2 | NEG | -85 | -62 | 25 | -10 |
| **PI 32:0** | 808,6 | 255,2 | NEG | -85 | -62 | 25 | -10 |
| **PI 34:2** | 832,6 | 253,2 | NEG | -85 | -62 | 25 | -10 |
| | | 281,3 | NEG | -85 | -62 | 25 | -10 |
| | | 253,2 | NEG | -85 | -62 | 25 | -10 |
| **PI 34:1** | 834,6 | 255,2 | NEG | -85 | -62 | 25 | -10 |
| | | 281,3 | NEG | -85 | -62 | 25 | -10 |
| | | 283,3 | NEG | -85 | -62 | 25 | -10 |
| **PI 34:0** | 836,6 | 255,2 | NEG | -85 | -62 | 25 | -10 |
| | | 283,3 | NEG | -85 | -62 | 25 | -10 |
| **PI 36:2** | 860,7 | 281,3 | NEG | -85 | -62 | 25 | -10 |
| **PI 36:1** | 862,7 | 281,3 | NEG | -85 | -62 | 25 | -10 |
| **PI 36:0** | 864,7 | 283,3 | NEG | -85 | -62 | 25 | -10 |
