# Supplementary material for: A Phospholipid Profile at 4 Months Predicts the Onset of Celiac Disease in at-Risk Infants
Source: Sci Rep. 2019 Oct 4;9:14303. doi: 10.1038/s41598-019-50735-7 (PMC6778072; doi:10.1038/s41598-019-50735-7)
Supplement: Supplementary file 6 — Supplemental Figures [file 41598_2019_50735_MOESM6_ESM.pdf]

A PHOSPHOLIPID PROFILE AT 4 MONTHS PREDICTS THE ONSET OF CELIAC DISEASE IN AT-RISK INFANTS

R. Auricchio[1,2], M. Galatola[1,2], D. Cielo[1,2], A. Amoresano[3], M. Caterino[4,5], E. De Vita[3], A. Illiano[3], R.Troncone[1,2], L. Greco[1,2]and M. Ruoppolo[4,5]

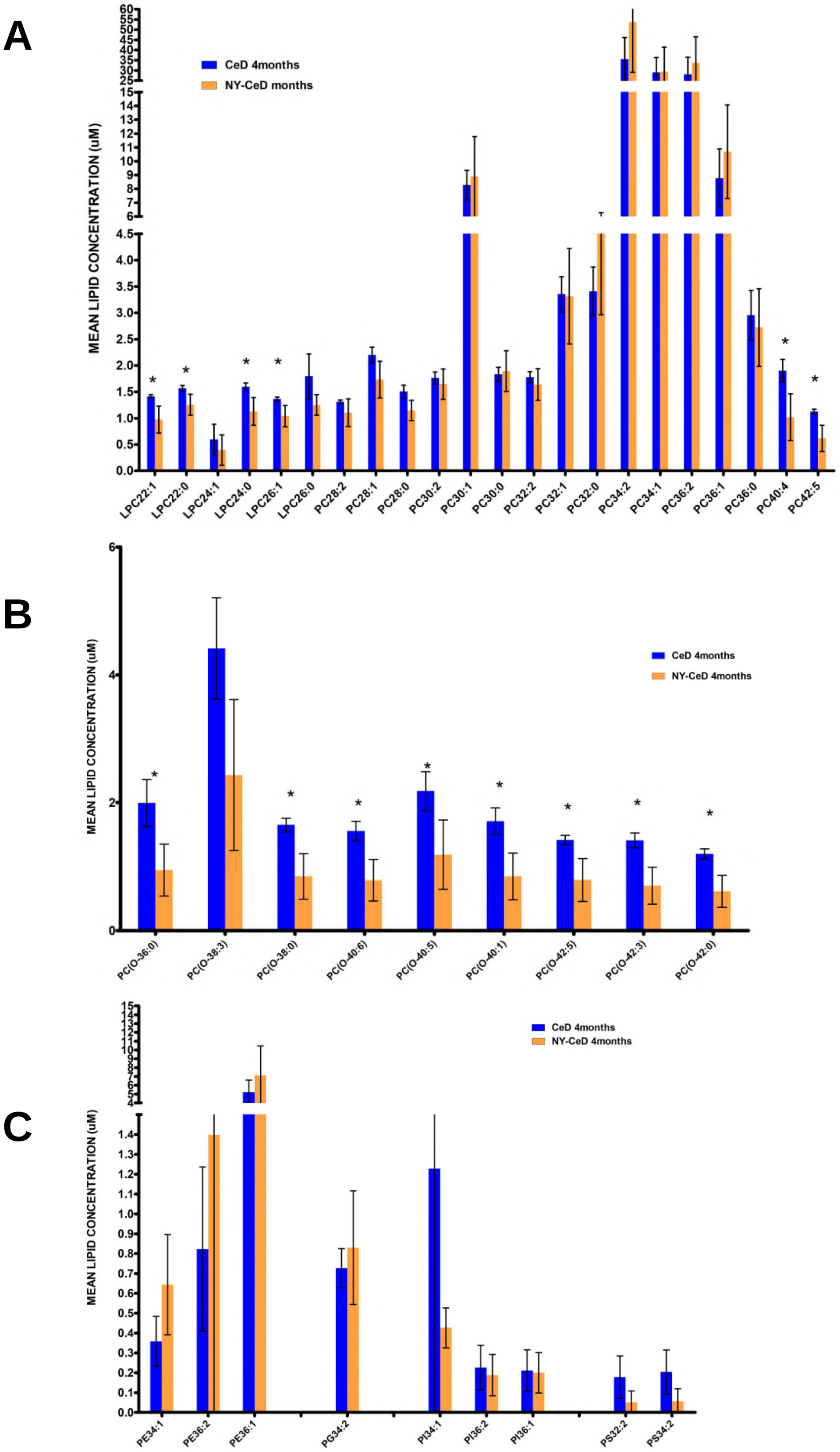
A
CeD 4months
NY-CeD months
MEAN LIPID CONCENTRATION (uM)
LPC22:1
LPC22:0
LPC24:1
LPC24:0
LPC26:1
LPC26:0
PC28:2
PC28:1
PC28:0
PC30:2
PC30:1
PC30:0
PC32:2
PC32:1
PC32:0
PC34:2
PC34:1
PC36:2
PC36:1
PC36:0
PC40:4
PC42:5
B
CeD 4months
NY-CeD 4months
MEAN LIPID CONCENTRATION (uM)
PC(O-36:0)
PC(O-38:3)
PC(O-38:0)
PC(O-40:6)
PC(O-40:5)
PC(O-40:1)
PC(O-42:5)
PC(O-42:3)
PC(O-42:0)
C
CeD 4months
NY-CeD 4months
MEAN LIPID CONCENTRATION (uM)
PE34:1
PE36:2
PE36:1
PG34:2
PI34:1
PI36:2
PI36:1
PS32:2
PS34:2

Figure 1S

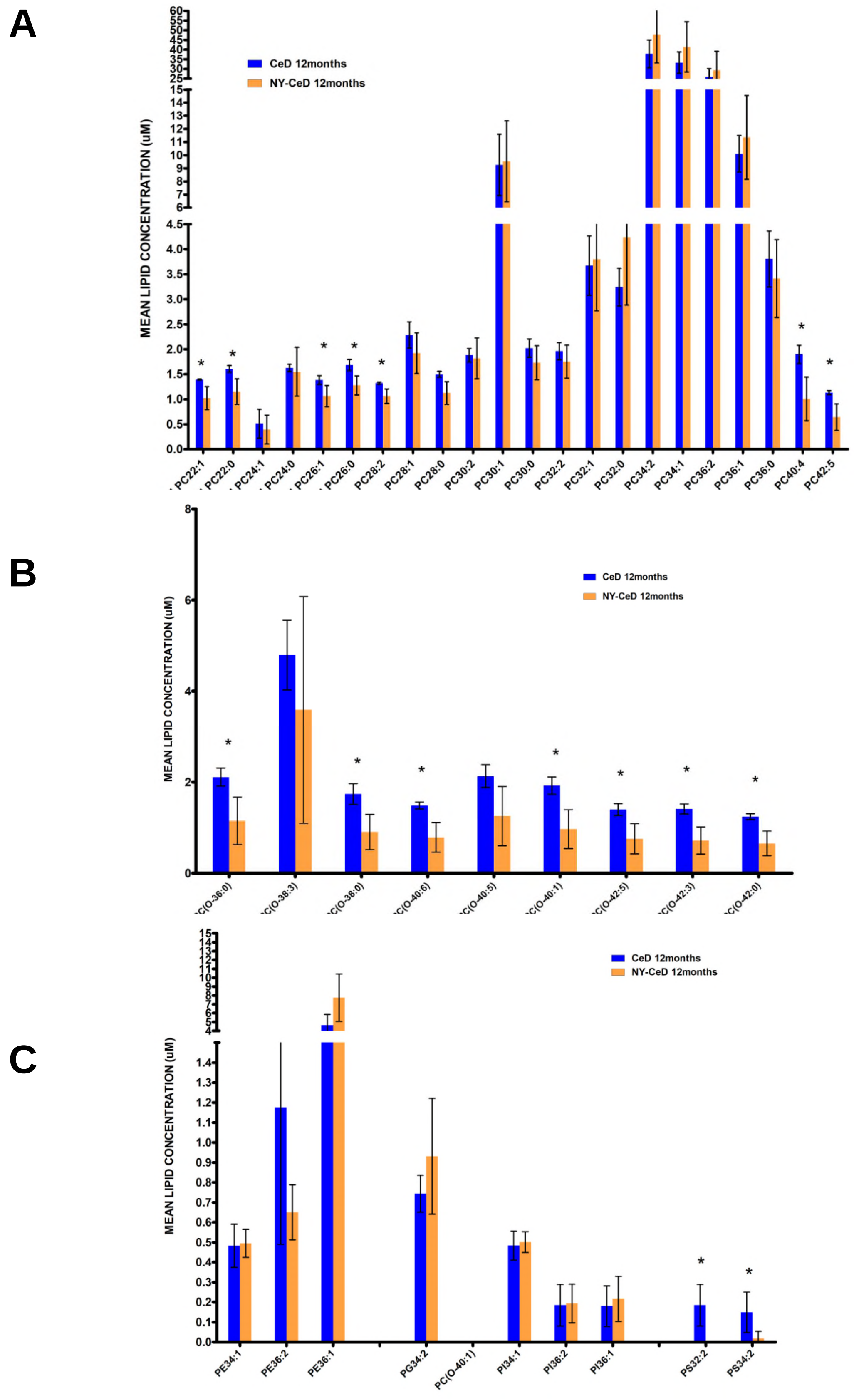
A
CeD 12months
NY-CeD 12months
MEAN LIPID CONCENTRATION (uM)
PC22:1
PC22:0
PC24:1
PC24:0
PC26:1
PC26:0
PC28:2
PC28:1
PC28:0
PC30:2
PC30:1
PC30:0
PC32:2
PC32:1
PC32:0
PC34:2
PC34:1
PC36:2
PC36:1
PC36:0
PC40:4
PC42:5
B
CeD 12months
NY-CeD 12months
MEAN LIPID CONCENTRATION (uM)
C
CeD 12months
NY-CeD 12months
MEAN LIPID CONCENTRATION (uM)
PE34:1
PE36:2
PE36:1
PG34:2
PC(O-40:1)
PI34:1
PI36:2
PI36:1
PS32:2
PS34:2

Figure 2S

Supplemental Figure 1S. The mean lipid concentration in CeD at 4 months in CeD *versus* NY-CeD. Lyso-phosphatidylcholine, LPC, phosphatidylcholine, PC, (Panel A); alkylacyl-phosphatidylcholine, PC-O, (PanelB); phosphatidyletanolamine, PE, phosphatidylglycerol, PG, phosphatidylinositol, PI, phosphatidylserine, PS, (Panel C) are shown.

Supplemental Figure 2S. The mean lipid concentration in CeD at 12 months in CeD *versus* NY-CeD. Lyso-phosphatidylcholine, LPC, phosphatidylcholine, PC, (Panel A); alkylacyl-phosphatidylcholine, PC-O, (PanelB); phosphatidyletanolamine, PE, phosphatidylglycerol, PG, phosphatidylinositol, PI, phosphatidylserine, PS, (Panel C) are shown. * Significant differences between the mean values, Bonferroni corrected.
